# Commentary: Arginine vasopressin receptor 1a is a therapeutic target for castration-resistant prostate cancer

**DOI:** 10.3389/fonc.2019.01490

**Published:** 2020-01-10

**Authors:** Giselle V. Ripoll, Marina Pifano, Juan Garona, Daniel F. Alonso

**Affiliations:** ^1^Laboratory of Molecular Oncology, Department of Science and Technology, National University of Quilmes, Bernal, Argentina; ^2^Scientific Investigator Career of National Scientific and Technical Research Council (CONICET), Buenos Aires, Argentina

**Keywords:** hormone-resistant cancer, vasopressin, relcovaptan, desmopressin, drug repurposing

Prostate cancer patients managed with androgen-deprivation therapy usually recur after a few years and the disease gradually becomes castration-resistant prostate cancer (CRPC). The role of tumor cell plasticity, including processes such as transdifferentiation and epithelial-mesenchymal transition, is pivotal in the development of androgen receptor (AR)-indifferent tumor variants (1). Cell plasticity may allow CRPC progression and metastasis by favoring reactivation of AR signaling as a result of different mechanisms of transcriptome reprogramming. Interestingly, dissection of such mechanisms can lead to the identification of novel vulnerabilities of aggressive tumor cells that can be targeted therapeutically. The recent work by Zhao et al. (2) identified the vasopressin receptor 1a (AVPR1a) as a critical effector in CRPC expressing the AR coactivator VAV3 and the constitutively active AR variant AR-V7. They demonstrated that ectopic expression of AVPR1a is capable of conferring castration resistance and agonist treatment with the receptor ligand, the natural hormone arginine vasopressin, activates ERK and CREB, signaling molecules known to promote prostate cancer progression. Interestingly, depletion of AVPR1a or inhibition by the selective AVPR1a antagonist relcovaptan resulted in decreased CRPC cell proliferation and reduced bone metastatic growth *in vivo*.

We completely agree with the authors in the sense that AVPR1a can be a potential target for CRPC therapy. We believe that clinical trials with relcovaptan are warranted, particularly in patients with bone-metastatic disease for which therapeutic options are limited. However, we want to point out that these results could be revealing other untapped antitumor properties of the vasopressin system-related drugs against prostate cancer cells. Our team has reported that the vasopressin analog desmopressin, a selective agonist for the vasopressin receptor 2 (AVPR2), significantly reduced tumor cell growth and migration in AR-negative CRPC (3). *In vitro* exposure to desmopressin also induced a dramatic decrease of the neuroendocrine markers chromogranin and neuron-specific enolase in aggressive CRPC cells (3). In prostate cancer, neuroendocrine transdifferentiation is known to be related with transition toward AR-indifference and metastatic phenotype. Besides, recent studies in orthotopic and heterotopic models of CRPC in athymic nude mice demonstrated an enhanced efficacy of docetaxel in combination with desmopressin (4, 5).

Agonist activation of AVPR2 present in various human cancer cell lines has been associated with triggering of antiproliferative signaling pathways involving canonical adenylate cyclase/cAMP/PKA axis activation. cAMP blocks the proliferation of many cell types, both normal and transformed, through multiple downstream effectors. It is known that increased cAMP levels inhibit the Raf/MAPK/ERK signaling pathway in a PKA-dependent manner, but this is not the only mitogenic pathway impaired by cAMP (6). Although cytostatic effect after AVPR2 stimulation is robust, the underlying mechanism is intriguing since AVPR2-mediated cell signaling may eventually lead to phosphorylation of hundreds of PKA substrates granting a complex and seemingly contradictory framework of signaling pathways as evidenced in immortalized epithelial cells after stimulation with desmopressin (7). In this sense, high cAMP levels and PKA activation may eventually lead to phosphorylation and activation of CREB. However, many factors contribute to final cell response. It is known that cAMP can either stimulate or inhibit tumor cell apoptosis, and PKA is able to mediate cAMP-promoted proapoptotic responses depending on the cell type and the cell context (8). In addition, it has been reported a crosstalk of cAMP/PKA that can inhibit RhoA-mediated signaling, thus affecting the aggressive behavior of CRPC cells (9). Taken together, it seems that CREB activation is not prominent after AVPR2 selective agonist action on cancer cells, being favored certain antiproliferative signals. In breast cancer cells expressing AVPR1a and AVPR2, natural vasopressin can activate both receptors but its affinity is higher for AVPR1a and the number of functional AVPR2 tends to be relatively low. Thus, vasopressin elicits AVPR1a-dependent proliferative signals mediated by ERK activation that clearly predominate over AVPR2-dependent antiproliferative signals (10). On the contrary, when AVPR1a is blocked by selective antagonists such as relcovaptan or tumor cells are exposed to specific AVPR2 agonists such as desmopressin, significant antiproliferative effects are achievable in hormone-resistant breast cancer cell lines (10, 11). Desmopressin also contributed to reduce aggressiveness of mammary tumors during chemotherapy in an immunocompetent mouse model (12). We have conducted a Phase 2 dose-escalation clinical trial of desmopressin as a perioperative adjuvant in patients with breast cancer (NCT01606072). Desmopressin appeared safe when administered in two slow infusions before and after surgery, and a rapid postoperative drop in circulating tumor cells was detected after treatment (13). In addition, we documented a reduced intraoperative bleeding associated to the well-known hemostatic effects of the compound (13).

From a wider perspective, the stimulating article by Zhao et al. (2) ratifies the relevance of the vasopressin system for searching novel therapeutic targets in hormone-resistant cancer and particularly in CRPC (Figure 1; see also Supplementary Table 1 for preclinical data summary). In this context, repurposing of already-used drugs with a non-oncology primary purpose stands as an interesting strategy to offer effective therapeutic options to cancer patients, allowing faster development and reducing safety concerns (14). The selective AVPR1a antagonist relcovaptan is a small-molecule inhibitor that has been safely and effectively used in clinical trials for Raynaud syndrome, dysmenorrhea and preterm labor. The specific AVPR2 agonist desmopressin is a synthetic peptide compound that has been employed for decades as an antidiuretic in the treatment of diabetic insipidus and enuresis, and as a hemostatic agent for the management of bleeding disorders, with a history of good tolerability and clinical effectiveness. Both compounds constitute promising therapeutic approaches for CRPC that deserve clinical testing either alone or in combination, as well as concurrently with standard chemotherapy regimens.

**Figure 1:**
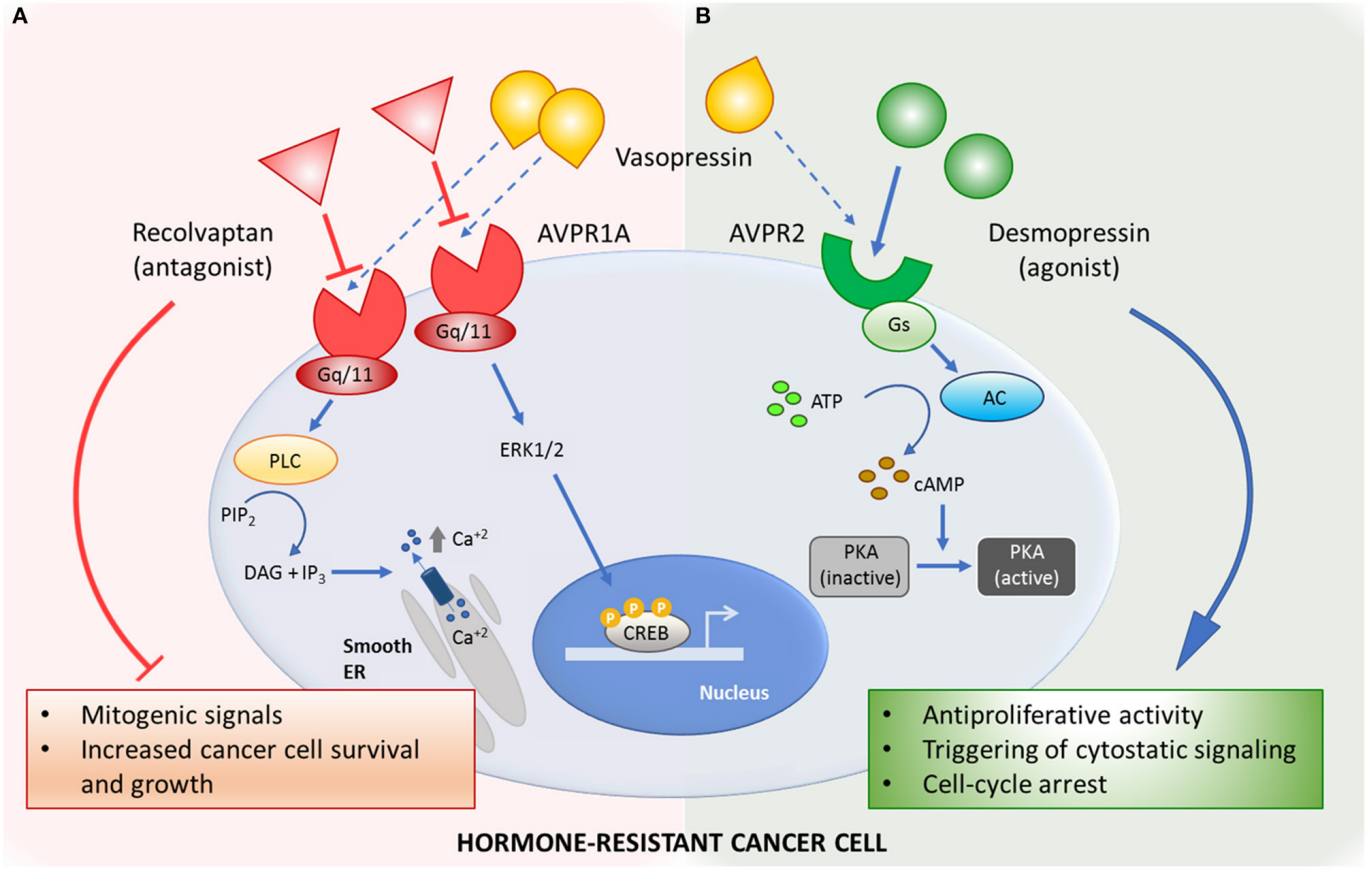
Mechanisms of action of the vasopressin system-related drugs in hormone-resistant cancer. **(A)** Selective AVPR1a-antagonist relcovaptan blocks mitogenic signals triggered by natural ligand vasopressin. **(B)** Activation of AVPR2 by selective agonist desmopressin inhibits cancer cell growth by activating antiproliferative signaling pathways. AC, adenylyl cyclase; ATP, adenosine triphosphate; AVPR1a, arginine vasopressin receptor type 1 A; AVPR2, arginine vasopressin receptor type 2; cAMP, cyclic adenosine monophosphate; CREB, cAMP-response-element-binding protein; DAG, diacylglycerol; ER, endoplasmic reticulum; ERK1/2, extracellular signal–regulated kinases; Gq/11, Gq protein alpha subunit; Gs, Gs protein alpha subunit; IP3, inositol-1,4,5-trisphosphate; PIP2, phosphatidyl inositol-bisphosphate; PKA, cAMP-dependent protein kinase; PLC, phospholipase C.

## Author Contributions

GR, MP, JG, and DA contributed to critical review of the literature and wrote the manuscript.

### Conflict of Interest

The authors declare that the research was conducted in the absence of any commercial or financial relationships that could be construed as a potential conflict of interest.
