# Supplementary material for: Commentary: Arginine vasopressin receptor 1a is a therapeutic target for castration-resistant prostate cancer
Source: Front Oncol. 2020 Jan 10;9:1490. doi: 10.3389/fonc.2019.01490 (PMC6970191; doi:10.3389/fonc.2019.01490)
Supplement: Supplementary file 1 [file Table_1.DOCX]

**Supplementary Table 1.** Repurposing opportunities for the vasopressin system-related drugs in hormone-resistant cancer. Preclinical data summary

| **Indication** | **Target** | **Drug** | **Mechanism of action** | **Cell line model** | **Reference** |
| --- | --- | --- | --- | --- | --- |
| Castration-resistant prostate cancer | AVPR1a | Relcovaptan^1^ | Antagonist | LNCaP, C4-2B | Zhao et al. (2) |
|  | AVPR2 | Desmopressin^2^ | Agonist | PC-3 | Pifano et al. (3) |
|  |  | Desmopressin | Agonist  (plus docetaxel) | LNCaP, PC-3 | Bass et al. (4)  Sasaki et al. (5) |
| Hormone-resistant breast cancer | AVPR1a | Relcovaptan | Antagonist | SKBR3 | Keegan et al. (6) |
|  | AVPR2 | Desmopressin | Agonist | MDA-MB-231, F3II^3^ | Garona et al. (7) |
|  |  | Desmopressin | Agonist  (plus paclitaxel or carmustine) | F3II | Ripoll et al. (8) |

^1^Non-peptidic synthetic compound (SR-49059). ^2^Peptidic synthetic vasopressin analog (1-deamino-8-D-arginine vasopressin or dDAVP). ^3^Mouse tumor cell line; all other cancer cell lines are human-derived.
